# Maximum Likelihood Estimation of Symmetric Group-Based Models via Numerical Algebraic Geometry

**DOI:** 10.1007/s11538-018-0523-2

**Published:** 2018-10-24

**Authors:** Dimitra Kosta, Kaie Kubjas

**Affiliations:** 1 School of Mathematics and Statistics, University of Glasgow, Glasgow, UK; 2 Department of Mathematics and Systems Analysis, Aalto University, Espoo, Finland

**Keywords:** Phylogenetics, Group-based models, Maximum likelihood estimation, Real algebraic geometry, Numerical algebraic geometry, Algebraic statistics

## Abstract

Phylogenetic models admit polynomial parametrization maps in terms of the root distribution and transition probabilities along the edges of the phylogenetic tree. For symmetric continuous-time group-based models, Matsen studied the polynomial inequalities that characterize the joint probabilities in the image of these parametrizations (Matsen in IEEE/ACM Trans Comput Biol Bioinform 6:89–95, 2009). We employ this description for maximum likelihood estimation via numerical algebraic geometry. In particular, we explore an example where the maximum likelihood estimate does not exist, which would be difficult to discover without using algebraic methods.

## Introduction

A phylogenetic tree is a rooted tree that depicts evolutionary relationships between species. A phylogenetic model is a statistical model describing the evolution of species on a phylogenetic tree. There is a discrete random variable associated with every vertex of the tree. The random variables associated with interior vertices are hidden and correspond to extinct species; the random variables associated with leaves are observed and correspond to extant species. The model parameters are the root distribution and the rate or transition matrices at the edges of the phylogenetic tree. There are different constraints on the model parameters depending on the phylogenetic model. The joint probabilities of random variables associated with leaves (leaf probabilities) are polynomials in the model parameters.

Cavender and Felsenstein (1987), and, separately, Lake (1987), introduced an algebraic approach to study phylogenetic models focusing on the search for phylogenetic invariants. A phylogenetic invariant of the model is a polynomial in the leaf probabilities which vanishes for every choice of model parameters. However, phylogenetic invariants alone do not describe the image of the parametrization map. One needs to include inequalities in order to obtain a complete description of the set of leaf probabilities corresponding to phylogenetic tree models.

This paper focuses on the study of continuous-time group-based models. In the rest of the paper, a phylogenetic model is always continuous-time unless written otherwise. Transition matrices of continuous-time phylogenetic models come from continuous-time Markov processes and they are matrix exponentials of rate matrices. Rate matrices of group-based models have a special structure that is determined by an abelian group. A *symmetric group-based model* assumes that the rate matrices along every edge are symmetric. In particular, a symmetric group-based model can be a submodel of a non-symmetric group-based model with extra symmetricity conditions on rate matrices. The precise definitions are given in Sect. 2.

Generating sets for phylogenetic invariants for group-based models are described in Sturmfels and Sullivant (2005), Casanellas et al. (2015). These papers consider discrete-time group-based models that require transition matrices to have a special structure determined by an abelian group, but they do not require transition matrices to be matrix exponentials of rate matrices. Generating sets derived in these papers are also valid under the continuous-time approach. However, inequalities defining both models differ, because the set of transition matrices is smaller under the continuous-time approach. A method for deriving the inequalities under the continuous-time approach is given in Matsen (Matsen 2009, Proposition 3.5). We will explicitly derive the semialgebraic description of the leaf probabilities of the CFN model on the tripod tree $K_{1,3}$.

Identifying the equation and inequality characterization of the leaf probabilities is only one part of the problem. The maximum likelihood estimation aims to find parameters that maximize the likelihood of observing the data for the given phylogenetic tree and phylogenetic model. Estimating the tree topology is another part of phylogenetic inference not considered here, see for example Dhar and Minin (2016) for a general overview on phylogenetic inference. Standard methods for the maximum likelihood estimation of the model parameters are the Newton–Raphson method (Schadt et al. 1998; Kenney and Gu 2012), quasi-Newton methods Olsen et al. (1994) and the EM algorithm (Felsenstein 1981; Friedman et al. 2002; Holmes and Rubin 2002; Hobolth and Jensen 2005). It is shown in Steel (1994), Chor et al. (2000) that likelihood functions on phylogenetic trees can have multiple local and global maxima, and thus none of the above methods can guarantee finding the global MLE as these methods are hill-climbing methods. It is stated in Dhar and Minin (2016) that currently no optimization method can guarantee to solve the optimization of the likelihood function over model parameters.

We suggest an alternative method that theoretically gives the solution to the maximum likelihood estimation problem with probability one. This method is based on numerical algebraic geometry (Sommese and Wampler 2005; Bates et al. 2013). The main idea behind this method is to use a numerical algebraic geometry package to compute all critical points of a likelihood function and then choose the critical point with the highest likelihood value. A similar method has been previously applied in optimal control (Rostalski et al. 2011) and in the life sciences (Gross et al. 2016).

Since phylogenetic models are not necessarily compact, the MLE might not even exist. We will use the proposed method to study an example for which the MLE does not exist for the CFN model on the tripod $K_{1,3}$ and a particular data vector. In this example, the global maximum is achieved when one of the model parameters goes to infinity. The nonexistence of the MLE would be very difficult to discover without the algebraic methods that we use in this paper, because standard numerical solvers output a solution close to the boundary of the model as we will demonstrate by solving the same MLE problem in Mathematica. One should see the example for the CFN model on the tripod $K_{1,3}$ as an illustration of a concept. It will be the subject of future work to develop a package that automatizes the computation in the phylogenetics setting, so that it can be easily used for studying further examples.

In Sect. 2, we introduce the preliminaries of phylogenetic models and present tools from Matsen (2009). Based on Matsen (2009), we state in Sect. 3 Proposition 3 that gives an algorithm for deriving the semialgebraic description of the leaf probabilities of a symmetric group-based model. A proof of Proposition 3 is given in “Appendix A”. Algorithm 1 in Sect. 4 outlines how to use numerical algebraic geometry to theoretically give the MLE with probability one. This algorithm is applied on the CFN model on the tripod in Example 5.

## Preliminaries of Group-Based Models

The exposition in this section largely follows Matsen (2009). A phylogenetic tree *T* is a rooted tree with *n* labeled leaves and it represents the evolutionary relationship between different species. Its leaves correspond to current species and the internal nodes correspond to common ancestors. There is a discrete random variable $X_v$ taking $k \in \mathbb {N}$ possible values associated to each vertex *v* of the tree *T*. Typical values for *k* are two, four or twenty, corresponding to a binary feature, the number of nucleotides and the number of amino acids. For example, if $k=4$, the random variable at a leaf represents the probability of observing *A*, *C*, *G* or *T* in the DNA of the species corresponding to the leaf.

A phylogenetic model assumes a collection of random variables under a Markov process (see Norris (1998) for a detailed introduction on Markov chains). The Markov process on the tree is determined entirely by the probability distribution at the root and the transition matrices $P^{(e)}$ associated to every edge *e* that reflect the change in the probabilities when moving from one vertex to another. The *transition matrices* have the form

$$\begin{aligned} P^{(e)}=\exp (t_e Q^{(e)}), \end{aligned}$$

where $\exp$ stands for matrix exponentiation, $t_e \ge 0$ represents time and $Q^{(e)}$ is a rate matrix. The non-diagonal entries of a *rate matrix* are nonnegative and each row sums to zero. In the rest of the paper, we assume that $t_e$ is incorporated in the rate matrix $Q^{(e)}$.

To define a *group-based phylogenetic model*, we first fix an abelian group $\mathcal {G}$, a finite set of labels $\mathcal {L}$ and a labeling function $L:\mathcal {G} \rightarrow \mathcal {L}$. Let $k=|\mathcal {G}|$. A rate matrix $Q^{(e)}$ is a rate matrix in the group-based model if it satisfies $Q^{(e)}_{g,h}=\psi ^{(e)}(h-g)$ for a vector $\psi ^{(e)} \in \mathbb {R}^{{\mathcal {G}}}$ with $\psi ^{(e)}(g_1)=\psi ^{(e)}(g_2)$ whenever $L(g_1)=L(g_2)$. Hence transition matrices of the group-based model form a subset of all the transition matrices that satisfy $P^{(e)}_{g,h}=f^{(e)}(h-g)$ for a probability vector $f^{(e)} \in \mathbb {R}^{{\mathcal {G}}}$ with $f^{(e)}(g_1)=f^{(e)}(g_2)$ whenever $L(g_1)=L(g_2)$. This is because the matrix exponentiation is defined as $e^M=\sum _{i=0}^{\infty } \frac{1}{i!} M^i$ and if a matrix *M* has the structure given by $\mathcal {G},\mathcal {L}$ and *L*, then one can check that also $M^i$ has the structure given by $\mathcal {G},\mathcal {L}$ and *L* for all $i \in \mathbb {N}$. The phylogenetic models we consider are symmetric, which means $Q^{(e)}_{g,h}=Q^{(e)}_{h,g}$. In the case of group-based models, this is equivalent to $L(g)=L(-g)$ for all $g \in \mathcal {G}$.

We will assume that the root distribution $\pi$ of a group-based model is uniform or the root distribution $\pi$ is such that the matrix $P \in \mathbb {R}^{\mathcal {G} \times \mathcal {G}}$ defined by $P_{g,h}:=\pi (h-g)$ is a transition matrix in the group-based model (i.e., it is exponential of a rate matrix in the group-based model). In the latter case, we add a new edge starting from the root and re-root the tree at the additional leaf. Instead of the previous root distribution, we use a new root distribution that puts all the mass at the identity and a new transition matrix which is the transition matrix *P* defined above. We will consider the new leaf as a hidden vertex while other leaves are considered as observed vertices. The same rerooting procedure is used in Sturmfels and Sullivant (2005), Matsen (2009). This approach does not allow completely arbitrary root distributions. In particular, a root distribution has to satisfy $\pi (g_1)=\pi (g_2)$ whenever $L(g_1)=L(g_2)$ and it has to satisfy inequalities that guarantee that the transition matrix *P* defined by $P_{g,h}:=\pi (h-g)$ is a matrix exponential of a rate matrix. The latter problem is called the embedding problem and is studied for $2 \times 2$ matrices in Kingman (1962) and for the Kimura 3-parameter model in Roca-Lacostena and Fernández-Sánchez (2017). In (Sturmfels and Sullivant (2005), Section 6), a workaround is described for deriving phylogenetic invariants for arbitrary root distributions for discrete-time group-based models. We will describe a workaround for deriving inequalities describing the CFN model for arbitrary root distributions; however, we do not know how to generalize this approach to other models.

The joint probability distributions $p_{i_1,\ldots ,i_n}=\text {Pr}(X_1=i_1,\ldots ,X_n=i_n)$ at the *n* leaves can be written as polynomials in the root probabilities and in the entries of the transition matrices. Denote by $\mathbf{p}$ the vector of joint probabilities $p_{i_1,\ldots ,i_n}$. As it is common in phylogenetic algebraic geometry, we will use the discrete Fourier transform for the groups $\mathcal {G}$ and $\mathcal {G}^n$ to study the set of transition matrices and the set of joint probabilities at the leaves for a given phylogenetic tree and a group-based model. The reason for this is that phylogenetic invariants are considerably simpler in the Fourier coordinates (see Sturmfels and Sullivant 2005).

Denote by $\hat{{\mathcal {G}}}$ the dual group of ${\mathcal {G}}$ whose elements are the group homomorphisms from ${\mathcal {G}}$ to the multiplicative group of complex numbers of magnitude one. Given a function $a:{\mathcal {G}} \rightarrow \mathbb {C}$, its *discrete Fourier transform* is the function $\check{a}: \hat{{\mathcal {G}}} \rightarrow \mathbb {C}$ defined by

$$\begin{aligned} \check{a}(\hat{g})=\sum _{h \in {\mathcal {G}}} \hat{g}(h) a(h). \end{aligned}$$

It is an invertible linear transformation given by the matrix *K*, where $K_{g,h}=\hat{g}(h)$. The group-based model being symmetric is equivalent to the vectors $\check{\psi }^{(e)}$ and $\check{f}^{(e)}$ being real, see (Matsen 2009, Section 2). If we regard the vector $\mathbf{p}$ of joint probabilities as a function of $\mathcal {G}^n$, i.e., as an element of ${{\mathrm{Hom}}}{(\mathcal {G}^n,\mathbb {C})}$, then the image of $\mathbf{p}$ under the Fourier transform of $\mathcal {G}^n$ is denoted $\mathbf{q}$.

The map from the entries of the rate matrices to the joint probabilities at leaves can be seen as a composition of four maps:

1 $$\begin{aligned} \{\psi ^{(e)}\}_{e \in E} \rightarrow \{\check{\psi }^{(e)}\}_{e \in E} \rightarrow \{\check{f}^{(e)}\}_{e \in E} \rightarrow \mathbf{q} \rightarrow \mathbf{p}. \end{aligned}$$

- The map from $\{\psi ^{(e)}\}_{e \in E}$ to $\{\check{\psi }^{(e)}\}_{e \in E}$ is given by the discrete Fourier transform of $\mathcal {G}$. It is an invertible linear transformation given by the matrix *K*.
- The map from $\{\check{\psi }^{(e)}\}_{e \in E}$ to $\{\check{f}^{(e)}\}_{e \in E}$ is given by
  2 $$\begin{aligned} \check{f}^{(e)}(g)=\exp (\check{\psi }^{(e)}(g)) \end{aligned}$$
  by (Matsen 2009, Lemma 2.2). It is an isomorphism between $\mathbb {R}^{E \times \mathcal {G}}$ and $\mathbb {R}_{>0}^{E \times \mathcal {G}}$.
- In the case when root distribution puts all the mass at the identity, the map from $\{\check{f}^{(e)}\}_{e \in E}$ to $\mathbf{q}$ is given by
  3 $$\begin{aligned} q_{\mathbf{g}}=\prod _{e \in E} \check{f}^{e}(^*g_e) \end{aligned}$$
  by (Székely et al. 1993, Theorem 3), where $^*g_e=\sum _{i \in \Lambda (e)} g_i$ and $\Lambda (e)$ is the set of observed leaves below *e*. See also (Sturmfels and Sullivant 2005, Sections 2 and 3) for a nice exposition of this result.
  In the case of the uniform root distribution, the identity (3) holds whenever $g_1+\cdots +g_n=0$. Otherwise $q_{\mathbf{g}}=0$. This follows from (Sturmfels and Sullivant 2005, Lemma 4 and formula (12)).
  On the domain $\mathbb {R}_{>0}^{E \times \mathcal {G}}$, this map is injective: (Matsen 2009, Proposition 3.3 and Proposition 3.4) give a map from $\mathbf{q}$ to $\{[\check{f}^{(e)}]^2\}_{e \in E}$. Taking nonnegative square roots results in a left inverse to the map (3).
- The map from $\mathbf{q}$ to $\mathbf{p}$ is given by the inverse of the discrete Fourier transform of $\mathcal {G}^n$. It is an invertible linear transformation given by the matrix $H^{-1}$, where *H* is the *n*-fold Kronecker product of the matrix *K*.

### Example 1

We will consider in detail the Cavender–Farris–Neyman (CFN) model (Cavender 1978; Farris 1973; Neyman 1971) on the rooted claw tree $T=K_{1,3}$. This example has been previously studied in (Sturmfels and Sullivant 2005, Example 3) and (Hosten et al. 2005, Example 14). The CFN model is a group-based model with $\mathcal {G}=\mathbb {Z}_2,\mathcal {L}=\{0,1\}$ and the labeling function *L* defined by $L(0)=0$ and $L(1)=1$. Denote the root distribution by $\pi =(\pi _0,\pi _1)$ and the transition matrices at edges $e_1,e_2,e_3$ by

$$\begin{aligned} P^{(e_1)} = \left( \begin{array}{ll} \alpha ^{e_1} &{} \beta ^{e_1} \\ \beta ^{e_1} &{} \alpha ^{e_1} \end{array} \right) , P^{(e_2)} = \left( \begin{array}{ll} \alpha ^{e_2} &{} \beta ^{e_2} \\ \beta ^{e_2} &{} \alpha ^{e_2}\end{array} \right) , P^{(e_3)} = \left( \begin{array}{ll} \alpha ^{e_3} &{} \beta ^{e_3} \\ \beta ^{e_3} &{} \alpha ^{e_3} \end{array} \right) . \end{aligned}$$

Since $\pi _i,\alpha ^{e_i},\beta ^{e_i}$ are probabilities, they are real numbers in [0, 1], $\pi _0+\pi _1=1$ and $\alpha ^{e_i}+\beta ^{e_i}=1$. Moreover, the restriction on the root distribution that it is uniform or defines a valid transition matrix in the CFN model gives $1 \ge \pi _0 \ge \frac{1}{2}$ and $\frac{1}{2} \ge \pi _1 \ge 0$; however, in Example 2 we will show that for the CFN model we can consider arbitrary root distributions. The determinant of $P^{(e_i)}$ is positive, because $P^{(e_i)}$ is the matrix exponential of a rate matrix $Q^{(e_i)}$. Conversely, for every $P^{(e_i)}$ satisfying these constraints, there exists a rate matrix $Q^{(e_i)}$ such that $P^{(e_i)}=\exp (t_{e_i}Q^{(e_i)})$ by (Kingman 1962, Proposition 2).

The joint probabilities at the leaves have the parametrization

$$\begin{aligned} p_{000} = \pi _0 \alpha ^{e_1} \alpha ^{e_2} \alpha ^{e_3} + \pi _1 \beta ^{e_1} \beta ^{e_2}\beta ^{e_3} ,&p_{001} = \pi _0 \alpha ^{e_1} \alpha ^{e_2} \beta ^{e_3} + \pi _1 \beta ^{e_1} \beta ^{e_2} \alpha ^{e_3},\\ p_{010} = \pi _0 \alpha ^{e_1} \beta ^{e_2} \alpha ^{e_3} + \pi _1 \beta ^{e_1} \alpha ^{e_2} \beta ^{e_3} ,&p_{011} = \pi _0 \alpha ^{e_1} \beta ^{e_2} \beta ^{e_3} + \pi _1 \beta ^{e_1} \alpha ^{e_2} \alpha ^{e_3},\\ p_{100} = \pi _0 \beta ^{e_1} \alpha ^{e_2} \alpha ^{e_3} + \pi _1 \alpha ^{e_1} \beta ^{e_2} \beta ^{e_3} ,&p_{101} = \pi _0 \beta ^{e_1} \alpha ^{e_2} \beta ^{e_3} + \pi _1 \alpha ^{e_1} \beta ^{e_2} \alpha ^{e_3},\\ p_{110} = \pi _0 \beta ^{e_1} \beta ^{e_2} \alpha ^{e_3} + \pi _1 \alpha ^{e_1} \alpha ^{e_2} \beta ^{e_3} ,&p_{111} = \pi _0 \beta ^{e_1} \beta ^{e_2} \beta ^{e_3} + \pi _1 \alpha ^{e_1} \alpha ^{e_2} \alpha ^{e_3}. \end{aligned}$$

In Sect. 3, we characterize this model in joint probabilities $p_{ijk}$ and without parameters $\pi _i,\alpha ^{e_i},\beta ^{e_i}$. This is called the implicit description of a model. It consists of polynomial equations and inequalities in $p_{ijk}$ that describe the joint probabilities that come from a parametrization by rate matrices. In the Fourier coordinates, these equations can always be chosen to be binomials for any group-based model and tree (Evans and Speed 1993; Székely et al. 1993). These binomials are characterized in (Sturmfels and Sullivant 2005, Theorem 1). In the case of the CFN model on $K_{1,3}$, these binomials are

$$\begin{aligned} \{ q_{001} q_{110} - q_{000} q_{111}, q_{010} q_{101} - q_{000} q_{111}, q_{100} q_{011} - q_{000} q_{111} \} \text {,} \end{aligned}$$

as was shown in (Sturmfels and Sullivant 2005, Example 3). The equations defining the model in the original coordinates can be obtained by applying the Fourier transformation of $(\mathbb {Z}_2)^3$ on these binomials:

$$\begin{aligned} q_{000}&= p_{000}+ p_{001}+ p_{010}+ p_{011}+ p_{100}+ p_{101}+ p_{110}+ p_{111}, \\ q_{001}&= p_{000}- p_{001}+ p_{010}- p_{011}+ p_{100}- p_{101}+ p_{110}- p_{111}, \\ q_{010}&=p_{000}+ p_{001}- p_{010}- p_{011}+ p_{100}+ p_{101}- p_{110}- p_{111}, \\ q_{011}&= p_{000}- p_{001}- p_{010}+ p_{011}+ p_{100}- p_{101}- p_{110}+ p_{111}, \\ q_{100}&= p_{000}+ p_{001}+ p_{010}+ p_{011}- p_{100}- p_{101}- p_{110}- p_{111}, \\ q_{101}&= p_{000}- p_{001}+ p_{010}- p_{011}- p_{100}+ p_{101}- p_{110}+ p_{111}, \\ q_{110}&= p_{000}+ p_{001}- p_{010}- p_{011}- p_{100}- p_{101}+ p_{110}+ p_{111}, \\ q_{111}&= p_{000}- p_{001}- p_{010}+ p_{011}- p_{100}+ p_{101}+ p_{110}- p_{111}. \end{aligned}$$

Finally, we introduce basic notions from commutative algebra and algebraic geometry. A good introduction is given in Cox et al. (1992). Let $R=\mathbb {R}[x_1,\ldots ,x_n]$ be a polynomial ring. A subset $I \subseteq R$ is an ideal if it is an additive subgroup of *R* and is closed under multiplication by elements of the ring. The radical of an ideal *I*, denoted by $\sqrt{I}$, consists of all the polynomials $f \in R$ such that some power $f^m$ of *f* is in *I*. Let *S* be a set of polynomials in *R* and let *k* be a field. In this article, *k* is always $\mathbb {R}$ or $\mathbb {C}$. The affine variety defined by *S* is

$$\begin{aligned} V(S)=\{(a_1,\ldots ,a_n) \in k^n: f(a_1,\ldots ,a_n)=0 \text { for all } f \in S \}. \end{aligned}$$

Let $\langle f_1,\ldots ,f_s \rangle$ be the ideal generated by $f_1,\ldots ,f_s$, i.e., the smallest ideal containing $f_1,\ldots ,f_s$. Then

$$\begin{aligned} V(f_1,\ldots ,f_s)=V(\langle f_1,\ldots ,f_s \rangle ). \end{aligned}$$

A point of the variety $V(f_1,\ldots ,f_s)$ is a smooth point if the Jacobian of $f_1,\ldots ,f_s$ has maximal possible rank. Otherwise a point of the variety is called singular. Let *T* be a subset of $k^n$. The Zariski closure $\overline{T}$ of *T* is the smallest affine variety containing *T*.

## Implicit Descriptions of Symmetric Group-Based Models

*Phylogenetic invariants* are polynomials that vanish at joint probabilities at leaves for a given model and tree. They were introduced in Cavender and Felsenstein (1987) and Lake (1987) and have been characterized for group-based phylogenetic models in (Sturmfels and Sullivant 2005, Theorem 1). *Phylogenetic varieties* are algebraic varieties derived from phylogenetic models and were first introduced in Allman and Rhodes (2003, 2004). In this paper, an algebraic variety is not necessarily irreducible. Phylogenetic invariants are elements of the ideal of a phylogenetic variety. Specifying a system of generators of the ideal of a phylogenetic variety is an important problem in phylogenetic algebraic geometry. However, the set of probability distributions forms only a (real, semialgebraic) subset of the phylogenetic variety, therefore providing a complete system of generators might have no biological interest. In Casanellas et al. (2015), a minimal set of phylogenetic invariants is constructed that defines the intersection of a phylogenetic variety with a Zariski open set. In the case of the Kimura 3-parameter model, all the leaf probabilities that are images of real parameters in the phylogenetic model (not in the complexification of the model) lie in this Zariski open set. The number of polynomials in this set is equal to the codimension of the phylogenetic variety and each polynomial has degree at most $|\mathcal {G}|$. This reduces drastically the number of phylogenetic invariants used: For the Kimura 3-parameter model on a quartet tree, it drops from 8002 generators of the ideal to the 48 polynomials described in (Casanellas and Fernández-Sánchez 2008, Example 4.9).

Besides phylogenetic invariants, polynomial inequalities are needed to give an exact characterization of joint probabilities at leaves for a given model and a tree. For general symmetric group-based models, polynomial inequalities that describe joint probabilities at leaves are studied in Matsen (2009). We recall (Matsen 2009, Propositions 3.3 and 3.4) that give the left inverse to the map (3) on the domain $\mathbb {R}^{E \times \mathcal {G}}_{>0}$.

### Proposition 1

(Matsen (2009), Proposition 3.3) Given some leaf edge *e*, let *i* denote the leaf vertex incident to *e* and let *v* be the internal vertex incident to *e*. Let *j*, *k* be leaf vertices different from *i* such that the path from *j* to *k* contains *v*. Let $w(g_i,g_j,g_k) \in \mathcal {G}^n$ assign state $g_x$ to leaf *x* for $x\in \{i,j,k\}$ and zero to all other leaf vertices. Then

$$\begin{aligned}{}[\check{f}^{(e)}(h)]^2=\frac{q_{w(h,-h,0)} q_{w(-h,0,h)}}{q_{w(0,-h,h)}}. \end{aligned}$$

### Proposition 2

(Matsen (2009), Proposition 3.4) Given some internal edge *e*, let the two vertices incident to *e* be *v* and $v'$. Let *i*, *j* (respectively, $i',j'$) be leaf vertices such that the path from *i* to *j* (respectively, the path from $i'$ to $j'$) contains *v* but not $v'$ (respectively, $v'$ but not *v*). Let $z(g_i,g_j,g_{i'},g_{j'}) \in \mathcal {G}^n$ assign state $g_x$ to leaf *x* for $x \in \{i,j,i',j'\}$ and zero to all other leaf vertices. Then

$$\begin{aligned}{}[\check{f}^{(e)}(h)]^2=\frac{q_{z(h,0,-h,0)} q_{z(0,-h,0,h)}}{q_{z(h,-h,0,0)} q_{z(0,0,-h,h)}}. \end{aligned}$$

The next proposition will summarize the procedure in Matsen (2009) to construct inequalities that describe joint probabilities. We will denote by $(K^{-1})_{g,:}$ the row of the matrix $K^{-1}$ labeled by *g* and by $(\check{f}^{(e)})^{(K^{-1})_{g,:}}$ the Laurent monomial $\prod _{h \in \mathcal {G}} (\check{f}^{(e)}(h))^{(K^{-1})_{g,h}}$.

### Proposition 3

Assume that the labeling function *L* satisfies $L(g)=L(-g)$ for all $g \in \mathcal {G}$. Consider the set of $\{\psi ^{(e)}\}_{e \in E}$ that satisfies $\sum _{g \in \mathcal {G}} \psi ^{(e)}(g)=0$, $\psi ^{(e)}(g_1)=\psi ^{(e)}(g_2)$ whenever $L(g_1)=L(g_2)$ and $\psi ^{(e)}(g) \ge 0$ for all nonzero $g \in \mathcal {G}$. The images of this set under the maps in (1) are:

(i) The constraints for $\{\check{\psi }^{(e)}\}_{e \in E}$ are obtained by substituting $\psi ^{(e)}$ by $K^{-1} \check{\psi }^{(e)}$ in the constraints for $\{\psi ^{(e)}\}_{e \in E}$. In particular, this gives $\check{\psi }^{(e)}(0)=0$, $(K^{-1} \check{\psi }^{(e)})(g_1)=(K^{-1} \check{\psi }^{(e)})(g_2)$ whenever $L(g_1)=L(g_2)$ and $(K^{-1} \check{\psi }^{(e)})(g) \ge 0$ for all nonzero $g \in \mathcal {G}$.
(ii) The constraints for $\{\check{f}^{(e)}\}_{e \in E}$ are $\check{f}^{(e)}(0)=1$, $(\check{f}^{(e)})^{(K^{-1})_{g_1,:}}=(\check{f}^{(e)})^{(K^{-1})_{g_2,:}}$ whenever $L(g_1)=L(g_2)$, $(\check{f}^{(e)})^{(K^{-1})_{g,:}} \ge 1$ for all nonzero $g \in \mathcal {G}$ and $\check{f}^{(e)}(g) > 0$ for all $g \in \mathcal {G}$. This equation and inequalities are equivalent to $\check{f}^{(e)}(0)=1$, $(\check{f}^{(e)})^{(K^{-1})_{g_1,:}}=(\check{f}^{(e)})^{(K^{-1})_{g_2,:}}$ whenever $L(g_1)=L(g_2)$, $(\check{f}^{(e)})^{2(K^{-1})_{g,:}} \ge 1$ for all nonzero $g \in \mathcal {G}$ and $\check{f}^{(e)}(g) > 0$ for all $g \in \mathcal {G}$. Here we have squared the inequalities $(\check{f}^{(e)})^{(K^{-1})_{g,:}} \ge 1$.
(iii) The constraints for $\mathbf{q}$ are given by phylogenetic invariants, equation $q_{00\ldots 0}=1$, inequalities $\mathbf{q} > 0$ and inequalities that are obtained by substituting expressions for $[\check{f}^{(e)}]^2$ in Propositions 1 and 2 to inequalities $(\check{f}^{(e)})^{2(K^{-1})_{g,:}} \ge 1$ in the previous item.
(iv) The constraints for $\mathbf{p}$ are obtained by substituting $\mathbf{q}$ by $H \mathbf{p}$ in the constraints for $\mathbf{q}$.

For the sake of completeness, a proof of Proposition 3 is given in “Appendix A”.

### Remark 1

In Proposition 3 item (iii), one applies Propositions 1 and 2 to obtain inequalities in the Fourier coordinates. However, in Propositions 1 and 2 one has a choice in choosing the leaf vertices. Since the Fourier coordinates are strictly positive, then any choice of leaf vertices in Propositions 1 and 2 gives equivalent inequalities in Proposition 3 item (iii) and it does not matter which choice we make.

### Example 2

We will derive the implicit description of the CFN model on the rooted claw tree $T=K_{1,3}$. We start with the case when $1 \ge \pi _0 > \frac{1}{2}$ and $\frac{1}{2} > \pi _1 \ge 0$. In addition to phylogenetic invariants in Example 1, applying Proposition 3 gives the following inequalities in Fourier coordinates:

4 $$\begin{aligned}&q_{000}=1,\nonumber \\&\mathbf{q} >0,\nonumber \\&\frac{q_{100} q_{010}}{q_{110}} \le 1, \frac{q_{110} q_{101}}{q_{011}} \le 1, \frac{q_{110} q_{011}}{q_{101}} \le 1, \frac{q_{101} q_{011}}{q_{110}} \le 1. \end{aligned}$$

The inequality $\frac{q_{100} q_{010}}{q_{110}} \le 1$ is for the hidden leaf corresponding to the root. Since $q_{000}=1$, we can multiply all the denominators by $q_{000}$ without changing the inequalities (4). Clearing denominators gives the following polynomial inequalities

$$\begin{aligned}&q_{000}=1,\\&\mathbf{q} >0,\\&q_{000} q_{110} - q_{100} q_{010} \ge 0, q_{000} q_{011} - q_{110} q_{101} \ge 0,\\&q_{000} q_{101} - q_{110} q_{011} \ge 0, q_{000} q_{110} - q_{101} q_{011} \ge 0. \end{aligned}$$

By applying the discrete Fourier transformation, we get the implicit description in the original coordinates

5 $$\begin{aligned}&p_{001}p_{010}-p_{000}p_{011}+p_{001}p_{100}-p_{000}p_{101}-p_{011}p_{110}-p_{101}p_{110}\nonumber \\&\quad +p_{010}p_{111}+p_{100}p_{111}=0, \end{aligned}$$

6 $$\begin{aligned}&p_{001}p_{010}-p_{000}p_{011}+p_{010}p_{100}-p_{011}p_{101}-p_{000}p_{110}-p_{101}p_{110}\nonumber \\&\quad +p_{001}p_{111}+p_{100}p_{111}=0,\end{aligned}$$

7 $$\begin{aligned}&p_{001}p_{100}+p_{010}p_{100}-p_{000}p_{101}-p_{011}p_{101}-p_{000}p_{110}-p_{011}p_{110}\nonumber \\&\quad +p_{001}p_{111}+p_{010}p_{111}=0,\end{aligned}$$

8 $$\begin{aligned}&p_{000} + p_{001} + p_{010} + p_{011} + p_{100} + p_{101} + p_{110} + p_{111} - 1=0,\end{aligned}$$

9 $$\begin{aligned}&p_{000} - p_{001} + p_{010} - p_{011} + p_{100} - p_{101} + p_{110} - p_{111} > 0,\end{aligned}$$

10 $$\begin{aligned}&p_{000} + p_{001} - p_{010} - p_{011} + p_{100} + p_{101} - p_{110} - p_{111} > 0,\end{aligned}$$

11 $$\begin{aligned}&p_{000} - p_{001} - p_{010} + p_{011} + p_{100} - p_{101} - p_{110} + p_{111} > 0,\end{aligned}$$

12 $$\begin{aligned}&p_{000} + p_{001} + p_{010} + p_{011} - p_{100} - p_{101} - p_{110} - p_{111} > 0,\end{aligned}$$

13 $$\begin{aligned}&p_{000} - p_{001} + p_{010} - p_{011} - p_{100} + p_{101} - p_{110} + p_{111} > 0,\end{aligned}$$

14 $$\begin{aligned}&p_{000} + p_{001} - p_{010} - p_{011} - p_{100} - p_{101} + p_{110} + p_{111} > 0,\end{aligned}$$

15 $$\begin{aligned}&p_{000} - p_{001} - p_{010} + p_{011} - p_{100} + p_{101} + p_{110} - p_{111} > 0,\end{aligned}$$

16 $$\begin{aligned}&-p_{010}p_{100}-p_{011}p_{100}-p_{010}p_{101}-p_{011}p_{101}+p_{000}p_{110}+p_{001}p_{110}\nonumber \\&\quad +p_{000}p _{111}+p_{001}p_{111} \ge 0,\end{aligned}$$

17 $$\begin{aligned}&-p_{001}p_{010}+p_{000}p_{011}+p_{000}p_{100}-p_{001}p_{101}-p_{010}p_{110}-p_{101}p_{110}\nonumber \\&\quad +p_{011}p _{111}+p_{100}p_{111} \ge 0,\end{aligned}$$

18 $$\begin{aligned}&p_{000}p_{010}-p_{001}p_{011}-p_{001}p_{100}+p_{0 00}p_{101}-p_{011}p_{110}-p_{100}p_{110}\nonumber \\&\quad +p_{010}p _{111}+p_{101}p_{111} \ge 0,\end{aligned}$$

19 $$\begin{aligned}&p_{000}p_{001}-p_{010}p_{011}-p_{010}p_{100}+p_{0 11}p_{101}-p_{100}p_{101}+p_{000}p_{110}\nonumber \\&\quad +p_{001}p _{111}+p_{110}p_{111} \ge 0. \end{aligned}$$

If $1 \ge \pi _1 > \frac{1}{2}$ and $\frac{1}{2} > \pi _0 \ge 0$, we can switch 0 and 1 and apply the previously considered case. We obtain the implicit description by switching 0 and 1 in the subindices of the equations and inequalities (5)–(19). This operation leaves all the equations and the inequalities (11), (13), (14), (16)–(19) the same. It changes the inequalities (9), (10), (12) and (15). Explicitly, the implicit description is

20 $$\begin{aligned}&p_{001}p_{010}-p_{000}p_{011}+p_{001}p_{100}-p_{000}p_{101}-p_{011}p_{110}-p_{101}p_{110}\nonumber \\&\quad +p_{010}p_{111}+p_{100}p_{111}=0,\end{aligned}$$

21 $$\begin{aligned}&p_{001}p_{010}-p_{000}p_{011}+p_{010}p_{100}-p_{011}p_{101}-p_{000}p_{110}-p_{101}p_{110}\nonumber \\&\quad +p_{001}p_{111}+p_{100}p_{111}=0,\end{aligned}$$

22 $$\begin{aligned}&p_{001}p_{100}+p_{010}p_{100}-p_{000}p_{101}-p_{011}p_{101}-p_{000}p_{110}-p_{011}p_{110}\nonumber \\&\quad +p_{001}p_{111}+p_{010}p_{111}=0,\end{aligned}$$

23 $$\begin{aligned}&p_{000} + p_{001} + p_{010} + p_{011} + p_{100} + p_{101} + p_{110} + p_{111} - 1=0,\end{aligned}$$

24 $$\begin{aligned}&p_{000} - p_{001} + p_{010} - p_{011} + p_{100} - p_{101} + p_{110} - p_{111} < 0,\end{aligned}$$

25 $$\begin{aligned}&p_{000} + p_{001} - p_{010} - p_{011} + p_{100} + p_{101} - p_{110} - p_{111} < 0,\end{aligned}$$

26 $$\begin{aligned}&p_{000} - p_{001} - p_{010} + p_{011} + p_{100} - p_{101} - p_{110} + p_{111} > 0,\end{aligned}$$

27 $$\begin{aligned}&p_{000} + p_{001} + p_{010} + p_{011} - p_{100} - p_{101} - p_{110} - p_{111} < 0,\end{aligned}$$

28 $$\begin{aligned}&p_{000} - p_{001} + p_{010} - p_{011} - p_{100} + p_{101} - p_{110} + p_{111} > 0,\end{aligned}$$

29 $$\begin{aligned}&p_{000} + p_{001} - p_{010} - p_{011} - p_{100} - p_{101} + p_{110} + p_{111} > 0,\end{aligned}$$

30 $$\begin{aligned}&p_{000} - p_{001} - p_{010} + p_{011} - p_{100} + p_{101} + p_{110} - p_{111} < 0,\end{aligned}$$

31 $$\begin{aligned}&-p_{010}p_{100}-p_{011}p_{100}-p_{010}p_{101}-p_{011}p_{101}+p_{000}p_{110}+p_{001}p_{110}\nonumber \\&\quad +p_{000}p _{111}+p_{001}p_{111} \ge 0,\end{aligned}$$

32 $$\begin{aligned}&-p_{001}p_{010}+p_{000}p_{011}+p_{000}p_{100}-p_{001}p_{101}-p_{010}p_{110}-p_{101}p_{110}\nonumber \\&\quad +p_{011}p _{111}+p_{100}p_{111} \ge 0,\end{aligned}$$

33 $$\begin{aligned}&p_{000}p_{010}-p_{001}p_{011}-p_{001}p_{100}+p_{0 00}p_{101}-p_{011}p_{110}-p_{100}p_{110}\nonumber \\&\quad +p_{010}p _{111}+p_{101}p_{111} \ge 0,\end{aligned}$$

34 $$\begin{aligned}&p_{000}p_{001}-p_{010}p_{011}-p_{010}p_{100}+p_{0 11}p_{101}-p_{100}p_{101}+p_{000}p_{110}\nonumber \\&\quad +p_{001}p _{111}+p_{110}p_{111} \ge 0. \end{aligned}$$

Finally, we consider the case when the root distribution is uniform. By (Sturmfels and Sullivant 2005, Proposition 31), one gets additional phylogenetic invariants

$$\begin{aligned} q_{100}=0, q_{010}=0, q_{001}=0 \text { and } q_{111}=0. \end{aligned}$$

Moreover, we have the following equation and inequalities:

$$\begin{aligned}&q_{000}=1,\\&q_{110}>0,q_{101}>0,q_{011}>0,\\&\frac{q_{110} q_{101}}{q_{011}} \le 1, \frac{q_{110} q_{011}}{q_{101}} \le 1, \frac{q_{101} q_{011}}{q_{110}} \le 1. \end{aligned}$$

In original coordinates, we get the following implicit description:

35 $$\begin{aligned}&p_{001}p_{010}-p_{000}p_{011}+p_{001}p_{100}-p_{000}p_{101}-p_{011}p_{110}-p_{101}p_{110}\nonumber \\&\quad +p_{010}p_{111}+p_{100}p_{111}=0,\end{aligned}$$

36 $$\begin{aligned}&p_{001}p_{010}-p_{000}p_{011}+p_{010}p_{100}-p_{011}p_{101}-p_{000}p_{110}-p_{101}p_{110}\nonumber \\&\quad +p_{001}p_{111}+p_{100}p_{111}=0,\end{aligned}$$

37 $$\begin{aligned}&p_{001}p_{100}+p_{010}p_{100}-p_{000}p_{101}-p_{011}p_{101}-p_{000}p_{110}-p_{011}p_{110}\nonumber \\&\quad +p_{001}p_{111}+p_{010}p_{111}=0,\end{aligned}$$

38 $$\begin{aligned}&p_{000} + p_{001} + p_{010} + p_{011} + p_{100} + p_{101} + p_{110} + p_{111} - 1=0,\end{aligned}$$

39 $$\begin{aligned}&p_{000} - p_{001} + p_{010} - p_{011} + p_{100} - p_{101} + p_{110} - p_{111} = 0,\end{aligned}$$

40 $$\begin{aligned}&p_{000} + p_{001} - p_{010} - p_{011} + p_{100} + p_{101} - p_{110} - p_{111} = 0,\end{aligned}$$

41 $$\begin{aligned}&p_{000} - p_{001} - p_{010} + p_{011} + p_{100} - p_{101} - p_{110} + p_{111} > 0,\end{aligned}$$

42 $$\begin{aligned}&p_{000} + p_{001} + p_{010} + p_{011} - p_{100} - p_{101} - p_{110} - p_{111} = 0,\end{aligned}$$

43 $$\begin{aligned}&p_{000} - p_{001} + p_{010} - p_{011} - p_{100} + p_{101} - p_{110} + p_{111} > 0,\end{aligned}$$

44 $$\begin{aligned}&p_{000} + p_{001} - p_{010} - p_{011} - p_{100} - p_{101} + p_{110} + p_{111} > 0,\end{aligned}$$

45 $$\begin{aligned}&p_{000} - p_{001} - p_{010} + p_{011} - p_{100} + p_{101} + p_{110} - p_{111} = 0,\end{aligned}$$

46 $$\begin{aligned}&-p_{001}p_{010}+p_{000}p_{011}+p_{000}p_{100}-p_{001}p_{101}-p_{010}p_{110}-p_{101}p_{110}\nonumber \\&\quad +p_{011}p _{111}+p_{100}p_{111} \ge 0,\end{aligned}$$

47 $$\begin{aligned}&p_{000}p_{010}-p_{001}p_{011}-p_{001}p_{100}+p_{0 00}p_{101}-p_{011}p_{110}-p_{100}p_{110}\nonumber \\&\quad +p_{010}p _{111}+p_{101}p_{111} \ge 0,\end{aligned}$$

48 $$\begin{aligned}&p_{000}p_{001}-p_{010}p_{011}-p_{010}p_{100}+p_{0 11}p_{101}-p_{100}p_{101}\nonumber \\&\quad +p_{000}p_{110}+p_{001}p _{111}+p_{110}p_{111} \ge 0. \end{aligned}$$

The *implicit description* of the CFN model on the tree $K_{1,3}$ for an arbitrary root distribution is given as the union of three sets: the set defined by equations and inequalities (5)–(19), the set defined by equations and inequalities (20)–(34) and the set defined by equations and inequalities (35)–(48).

### Remark 2

Identifiability of parameters of a phylogenetic model means that if for a fixed tree two sets of parameters map to the same joint probabilities at leaves, then these sets of parameters must be equal. Generic identifiability means that this statement is true with probability one. The identifiability of the CFN model was shown in (Hendy 1991, Theorem 1), of the Kimura 3-parameter model in (Steel et al. 1998, Theorem 7) and the generic identifiability of the general Markov model in Chang (1996). The identifiability of any group-based model follows also from the proof of Proposition 3, since each of the maps in (1) is an isomorphism in the region we are interested in.

### Corollary 1

Consider a symmetric group-based model. Any $\mathbf{p}$ satisfying the equations and inequalities described in Proposition 3 that satisfies one of the inequalities with equality comes from a parametrization with an off-diagonal zero in the rate matrix $Q^{(e)}$ for some $e \in E$.

### Proof

There are two different kinds of inequalities in item (4) of Proposition 3. The strict inequalities can never be satisfied with equality. The non-strict inequalities in each step are obtained by substituting the inverse map to the inequalities in the previous step. Hence $\mathbf{p}$ satisfies one of the non-strict inequalities with equality if and only if it has a preimage $\{\psi ^{(e)}\}_{e \in E}$ that satisfies one of the inequalities $\psi ^{(e)}(g) \ge 0$ with equality. $\square$

### Example 3

We consider the CFN model. A joint probability vector $\mathbf{p}$ satisfying the assumptions of Corollary 1 has in its parametrization the rate matrix $$Q^{(e)}=\begin{pmatrix} 0 &{} 0\\ 0 &{} 0 \end{pmatrix}$$ for some $e \in E$. The transition matrix corresponding to the same edge is $$P^{(e)}=\begin{pmatrix} 1 &{} 0\\ 0 &{} 1 \end{pmatrix}$$.

## Maximum Likelihood Estimation via Numerical Algebraic Geometry

In this section, we use the terminology and notation introduced in Sect. 2. In particular, $p_{i_1,\ldots ,i_n}$ are the joint probability distributions at the *n*-leaves. Let $\mathbf{u}=(u_{i_1,\ldots ,i_n})_{(i_1,\ldots ,i_n) \in \mathcal {G}^n}$ be a vector of observations at leaves. The log-likelihood function of a phylogenetic model is

$$\begin{aligned} l_{\mathbf{u}}(\mathbf{p})=\sum _{(i_1,\ldots ,i_n) \in \mathcal {G}^n} u_{i_1,\ldots ,i_n} \log p_{i_1,\ldots ,i_n}. \end{aligned}$$

Maximum likelihood estimation aims to find a vector of joint probability distributions at leaves or model parameters (if the joint probabilities are considered as polynomials in model parameters) that lies in the model and maximizes the log-likelihood function for a given observation $\mathbf{u}$.

### Example 4

In (Hosten et al. 2005, Example 14), maximum likelihood estimation on the Zariski closure of the CFN model on $K_{1,3}$ is considered. This is the model that is defined by the equations in Example 2. For generic data, the number of complex critical points of the likelihood function on the Zariski closure of a model is called the *ML degree*. It is shown in (Hosten et al. 2005, Example 14) that the ML degree of the CFN model on $K_{1,3}$ is 92. Using tools from numerical algebraic geometry, one can compute the 92 critical points and among the real critical points choose the one that gives the maximal value of the log-likelihood function.

However, the MLE can lie on the boundary of a statistical model or even not exist. Neither of this can be detected by considering only the Zariski closure of the model. We will see the latter happening for the CFN model on $K_{1,3}$ in Example 5.

In practice, the MLE is solved using numerical methods such as the Newton–Raphson method (Schadt et al. 1998; Kenney and Gu 2012), quasi-Newton methods Olsen et al. (1994) and the EM algorithm (Felsenstein 1981; Friedman et al. 2002; Holmes and Rubin 2002; Hobolth and Jensen 2005). However, since these methods are hill-climbing methods and the likelihood function on phylogenetic trees can have multiple local maxima (Steel 1994; Chor et al. 2000), they are only guaranteed to give a local maximum or a saddle point of the log-likelihood function and not necessarily the global maximum. Usually one uses a heuristic to find a good initialization for these methods or runs them for different starting points and chooses the output that maximizes the log-likelihood function.

We suggest a global method based on numerical algebraic geometry that theoretically gives the solution to the maximum likelihood estimation problem on phylogenetic trees with probability one. The main idea behind numerical algebraic geometry is homotopy continuation. Homotopy continuation finds isolated complex solutions of a system of polynomial equations starting from the known solutions of another system of polynomial equations. Numerical algebraic geometry methods give theoretically correct results with probability one, meaning that bad phenomena can happen when certain parameters are chosen from a measure zero set. An introduction to numerical algebraic geometry can be found in Sommese and Wampler (2005), Bates et al. (2013). In our context, the system of polynomial equations that we wish to solve comes from the Karush–Kuhn–Tucker (KKT) conditions (Karush 1939; Kuhn and Tucker 1951) for the optimization problem that maximizes the likelihood function on a phylogenetic model. The set of solutions of this polynomial system contains all the critical points of the likelihood function. The global maximum of the likelihood function is the solution of the polynomial system that maximizes the likelihood function among all the solutions that lie in the model.

This global approach for solving a nonconvex optimization problem on a set that is described by polynomial equations and inequalities has been previously employed in optimal control Rostalski et al. (2011) and in the life sciences (Gross et al. 2016). Our setup and algorithm are similar to those in Rostalski et al. (2011), although we provide further lemmas that allow us to decompose the system of polynomial equations that we want to solve to simpler systems of polynomial equations. The article Gross et al. (2016) uses Fritz John conditions instead of KKT conditions and focuses mostly on optimization problems on sets that are described by polynomial equations only. Sets that are described by polynomial equations and inequalities are considered in Section 3 of the supplementary material of Gross et al. (2016). In particular, the ideas for Theorem 1 and Remark 3 appear there.

More specifically, consider the optimization problem

49 $$\begin{aligned}&\max F(x) \nonumber \\&\text {subject to} \nonumber \\&\qquad G_i(x) \ge 0 \text { for } i=1,\ldots ,m, \nonumber \\&\qquad H_j(x)=0 \text { for } j=1,\ldots ,l. \end{aligned}$$

If $x^*$ is a local optimum and the optimization problem satisfies first-order constraint qualifications, then there exist $\mu _i$, where $i=1,\ldots ,m$, and $\lambda _j$, where $j=1,\ldots ,l$, such that $x^*$ satisfies the *KKT conditions*:

50 $$\begin{aligned}&-\nabla F(x)+\sum _{i=1}^m \mu _i \nabla G_i(x)+\sum _{j=1}^l \lambda _j \nabla H_j(x)=0, \end{aligned}$$

51 $$\begin{aligned}&G_i(x) \ge 0 \text { for } i=1,\ldots ,m,\end{aligned}$$

52 $$\begin{aligned}&H_j(x)=0 \text { for } j=1,\ldots ,l,\end{aligned}$$

53 $$\begin{aligned}&\mu _i \ge 0 \text { for } i=1,\ldots ,m,\end{aligned}$$

54 $$\begin{aligned}&\mu _i G_i(x)=0 \text { for } i=1,\ldots ,m. \end{aligned}$$

One first-order constraint qualification is the *constant rank constraint qualification (CRCQ)* defined in Janin (1984). A point satisfies the CRCQ if there is a neighborhood of the point where gradients of the equations and gradients of the active inequalities, i.e., inequalities that the point satisfies with equality, have constant rank.

We also consider the optimization problem

55 $$\begin{aligned}&\max F(x) \nonumber \\&\text {subject to} \nonumber \\&\qquad H_j(x)=0 \text { for } j=1,\ldots ,l. \end{aligned}$$

If $x^*$ is a local optimum of the optimization problem (55), then there exist $\lambda _j$, where $j=1,\ldots ,l$, such that $x^*$ satisfies the *Lagrange conditions*:

56 $$\begin{aligned}&-\nabla F(x)+\sum _{j=1}^l \lambda _j \nabla H_j(x),\end{aligned}$$

57 $$\begin{aligned}&H_j(x) \text { for } j=1,\ldots ,l. \end{aligned}$$

In the rest of the section, we assume that the KKT conditions (50)–(54) and the Lagrange conditions (56)–(57) are polynomial. In this case, a point satisfies the CRCQ if it is a smooth point of the variety defined by the equations and active inequalities.

Let $L \subseteq \mathbb {C}[\mu ,\lambda ,x]$ be the ideal generated by the polynomials on the left-hand sides of the equations (50), (52) and (54) in the KKT conditions. For $S \subseteq [m]$, let $L_S \subseteq \mathbb {C}[\mu _S,\lambda ,x]$ be the ideal generated by the polynomials in the Lagrange conditions for the optimization problem

$$\begin{aligned}&\max F(x)\\&\text {subject to}\\&\qquad G_i(x)=0 \text { for } i \in S,\\&\qquad H_j(x)=0 \text { for } j=1,\ldots ,l. \end{aligned}$$

Specifically, let $L_S \subseteq \mathbb {C}[\mu _S,\lambda ,x]$ be generated by the polynomials

$$\begin{aligned}&-\nabla F(x)+\sum _{i\in S} \mu _i \nabla G_i(x)+\sum _{j=1}^l \lambda _j \nabla H_j(x),\\&G_i(x) \text { for } i\in S,\\&H_j(x) \text { for } j=1,\ldots ,l. \end{aligned}$$

We denote by $I_S \subseteq \mathbb {C}[x]$ the ideal generated by the constraints in the above optimization problem, i.e., $I_S=\langle G_i, H_j: i \in S, j=1,\ldots ,l \rangle$.

### Theorem 1

Let *L* and $L_S$ be as defined above. Then

$$\begin{aligned} V\left( L \cap \mathbb {C}[x]\right) =\bigcup _{S \subseteq [m]} V\left( L_S \cap \mathbb {C}[x]\right) . \end{aligned}$$

The idea behind Theorem 1 is that instead of optimizing a function over a semialgebraic set, one can optimize the function over the Zariski closure of the semialgebraic set and the Zariski closures of each of the boundaries of the semialgebraic set. This concept is discussed in Section 3 of the supplementary material of Gross et al. (2016).

### Proof

First take an element $(\mu , \lambda , x)$ of *V*(*L*). Let *S* be such that $G_i(x)=0$ for all $i \in S$. Then $(\mu _S, \lambda , x) \in V(L_S)$, where $\mu _S$ is the projection of $\mu$ to the coordinates in *S*. Conversely, let $(\mu _S, \lambda , x) \in V(L_S)$. Let $\mu \in \mathbb {C}^m$ be such that $\mu _i=(\mu _S)_i$ for $i\in S$ and $\mu _i=0$ otherwise. Then $(\mu , \lambda , x) \in V(L)$.

We have shown that $\pi _x (V(L))=\cup \pi _x(V(L_S))$, where $\pi _x$ is the projection of $(\mu , \lambda , x)$ or $(\mu _S, \lambda , x)$ on *x*. By the Closure Theorem (Cox et al. 1992, Theorem 3.2.3), $V(L \cap \mathbb {C}[x])$ is the smallest algebraic variety containing $\pi _x (V(L))$ and $V(L_S \cap \mathbb {C}[x])$ is the smallest algebraic variety containing $\pi _x(V(L_S))$. The inclusion $V(L \cap \mathbb {C}[x]) \subseteq \cup V(L_S \cap \mathbb {C}[x])$ holds, because the right-hand side is a variety and contains $\cup \pi _x(V(L_S))$ and hence $\pi _x (V(L))$. On the other hand, since $\pi _x(V(L_S)) \subseteq \pi _x (V(L))$ for every *S*, also $V(L_S \cap \mathbb {C}[x]) \subseteq V(L \cap \mathbb {C}[x])$ for every *S*. Hence $V(L \cap \mathbb {C}[x])=\cup V(L_S \cap \mathbb {C}[x])$. $\square$

Theorem 1 suggests Algorithm 1 for solving the equations in the KKT conditions. Algorithm 1 is related to (Rostalski et al. 2011, Algorithm 1) and (Gross et al. 2016, Algorithm 3). 
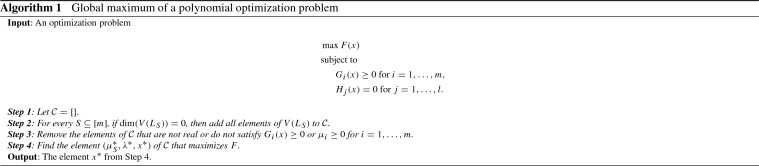


### Corollary 2

If *V*(*L*) is finite and the global maxima of the optimization problem (49) satisfy CRCQ, then Algorithm 1 outputs the global maxima.

### Proof

Theorem 1 implies that $V(L \cap \mathbb {C}[x])=\cup V(L_S \cap \mathbb {C}[x])$. The variety *V*(*L*) being finite implies that $V(L \cap \mathbb {C}[x])$ and hence all $V(L_S \cap \mathbb {C}[x])$ are finite. Hence after Step 2, the list $\mathcal {C}$ contains all solutions of Eqs. (50), (52) and (54) in the KKT conditions. Since the global maxima satisfy the CRCQ, they must be solutions of these equations. By choosing among the real solutions that satisfy inequalities (51) and (53) in the KKT conditions the ones that maximize the value of the cost function *F*, we get the global maxima. $\square$

We are interested in the optimization problem, when the cost function is the log-likelihood function $l_\mathbf{u }(\mathbf p )=\sum u_{i_1,\ldots ,i_n} \log p_{i_1,\ldots ,i_n}$ and the constraints are polynomial equations and inequalities that describe a statistical model (written as $H_j(\mathbf p )=0$ for $j=1,\ldots ,l$ and $G_i(\mathbf p )\ge 0$ for $i=1,\ldots ,m$, respectively). Although Eq. (50) is not polynomial for $F=l_\mathbf{u}$, it can be made polynomial by multiplying the equation

$$\begin{aligned} \frac{\partial l_\mathbf{u}(\mathbf p )}{\partial p_{i_1,\ldots ,i_n}}=\sum _{i=1}^m \mu _i \frac{\partial G_i(\mathbf p )}{\partial p_{i_1,\ldots ,i_n}} + \sum _{j=1}^l \lambda _j \frac{\partial H_j(\mathbf p )}{\partial p_{i_1,\ldots ,i_n}} \end{aligned}$$

with the variable $p_{i_1,\ldots ,i_n}$.

One of the reasons why the variety $V(L_S)$ in Step 2 of Algorithm 1 might not be finite is that the Lagrange conditions for MLE might be satisfied by higher-dimensional components where some variable is identically zero. For MLE, Gross and Rodriguez have defined a modification of the Lagrange conditions, known as Lagrange likelihood equations (Gross and Rodriguez 2014, Definition 2), whose solution set does not contain solutions with some variable equal to zero if the original data does not contain zeros (Gross and Rodriguez 2014, Proposition 1). However, the Lagrange likelihood equations can be applied only to homogeneous prime ideals. This motivates us to study Lagrange conditions for decompositions of ideals.

### Lemma 1

Assume that the ideal $J=\langle G_i: i = 1,\ldots , m \rangle$ decomposes as $J=J_1 \cap J_2$, where $J_1=\langle G^{(1)}_j: j = 1,\ldots , m_1 \rangle$ and $J_2=\langle G^{(2)}_k: k = 1,\ldots , m_2 \rangle$. If $x^*$ satisfies the Lagrange conditions for the optimization problem max *F*(*x*) subject to $G_i(x)=0$ for $i=1,\ldots ,m$, then $x^*$ satisfies the Lagrange conditions for the optimization problem max *F*(*x*) subject to $G^{(1)}_j(x)=0$ for $j=1,\ldots ,m_1$ or for the optimization problem max *F*(*x*) subject to $G^{(2)}_k(x)=0$ for $k=1,\ldots ,m_2$.

### Proof

Since $J=J_1 \cap J_2$, we have $J=\langle G^{(1)}_j G^{(2)}_k: j=1,\ldots ,m_1,k=1,\ldots ,m_2 \rangle$. Hence the optimization problem max *F*(*x*) subject to $G_i(x)=0$ for $i=1,\ldots ,m$ is equivalent to max *F*(*x*) subject to $G^{(1)}_j G^{(2)}_k(x)=0$ for $j=1,\ldots ,m_1,k=1,\ldots ,m_2$. The Lagrange conditions for the latter optimization problem are

$$\begin{aligned} \frac{\partial F}{\partial x}&= \sum _{j,k} \lambda _{jk} \left( \frac{\partial G^{(1)}_j}{\partial x} G^{(2)}_k + \frac{\partial G^{(2)}_k}{\partial x} G^{(1)}_j\right) \\&=\sum _{j} \frac{\partial G^{(1)}_j}{\partial x} \left( \sum _k \lambda _{jk} G^{(2)}_k\right) +\sum _{k} \frac{\partial G^{(2)}_k}{\partial x} \left( \sum _j \lambda _{jk} G^{(1)}_j\right) ,\\&G^{(1)}_j G^{(2)}_k=0 \text { for } j=1,\ldots ,m_1,k=1,\ldots ,m_2. \end{aligned}$$

If there exists *k* such that $G^{(2)}_k(x^*) \ne 0$, then we must have $G^{(1)}_j(x^*)=0$ for $j=1,\ldots ,m_1$. Hence $x^*$ satisfies

$$\begin{aligned} \frac{\partial F}{\partial x}&=\sum _{j} \frac{\partial G^{(1)}_j}{\partial x} \left( \sum _k \lambda _{jk} G^{(2)}_k\right) +\sum _{k} \frac{\partial G^{(2)}_k}{\partial x} \left( \sum _j \lambda _{jk} G^{(1)}_j\right) \\&=\sum _{j} \frac{\partial G^{(1)}_j}{\partial x} \left( \sum _k \lambda _{jk} G^{(2)}_k\right) ,\\&G^{(1)}_j=0 \text { for } j=1,\ldots ,m_1. \end{aligned}$$

Defining $\lambda ^{(1)}_j=\sum _k \lambda _{jk} G^{(2)}_k$, we see that $x^*$ satisfies Lagrange conditions for the optimization problem max *F*(*x*) subject to $G^{(1)}_j(x)=0$ for $j=1,\ldots ,m_1$. Otherwise $G^{(2)}_k(x^*)=0$ for $k=1,\ldots ,m_2$ and $x^*$ satisfies Lagrange conditions for the optimization problem max *F*(*x*) subject to $G^{(2)}_k(x)=0$ for $k=1,\ldots ,m_2$. $\square$

### Lemma 2

Let $J=J_1 \cap J_2$ and $K=K_1 \cap K_2$. If $x^*$ satisfies the Lagrange conditions for the optimization problem max *F*(*x*) subject to the generators of $J+K$, then $x^*$ satisfies the Lagrange conditions for one of the optimization problems max *F*(*x*) subject to the generators of $J_j+K_k$, where $j,k \in \{1,2\}$.

### Proof

Assume $J_1=\langle G^{(1)}_j: j = 1,\ldots , m_1 \rangle$, $J_2=\langle G^{(2)}_k: k = 1,\ldots , m_2 \rangle$, $K_1=\langle H^{(1)}_j: j = 1,\ldots , n_1 \rangle$ and $K_2=\langle H^{(2)}_k: k = 1,\ldots , n_2 \rangle$. Then $J=\langle G^{(1)}_j G^{(2)}_k: j=1,\ldots ,m_1,k=1,\ldots ,m_2 \rangle$ and $K=\langle H^{(1)}_j H^{(2)}_k: j=1,\ldots ,n_1,k=1,\ldots ,n_2 \rangle$. The Lagrange conditions for the generators of $J+K$ are

$$\begin{aligned} \frac{\partial F}{\partial x}&= \sum _{j} \frac{\partial G^{(1)}_j}{\partial x} \left( \sum _k \lambda _{jk} G^{(2)}_k\right) +\sum _{k} \frac{\partial G^{(2)}_k}{\partial x} \left( \sum _j \lambda _{jk} G^{(1)}_j\right) \\&\quad +\, \sum _{j} \frac{\partial H^{(1)}_j}{\partial x} \left( \sum _k \mu _{jk} H^{(2)}_k\right) +\sum _{k} \frac{\partial H^{(2)}_k}{\partial x} \left( \sum _j \mu _{jk} H^{(1)}_j\right) ,\\&G^{(1)}_j G^{(2)}_k =0 \text { for } j=1,\ldots ,m_1,k=1,\ldots ,m_2,\\&H^{(1)}_j H^{(2)}_k =0 \text { for } j=1,\ldots ,n_1,k=1,\ldots ,n_2. \end{aligned}$$

If there exists $k_1$ such that $G^{(2)}_{k_1}(x^*) \ne 0$ and $k_2$ such that $H^{(2)}_{k_2}(x^*) \ne 0$, then we must have $G^{(1)}_j(x^*)=0$ for $j=1,\ldots ,m_1$ and $H^{(1)}_j(x^*)=0$ for $j=1,\ldots ,n_1$. Hence $x^*$ satisfies

$$\begin{aligned} \frac{\partial F}{\partial x}&= \sum _{j} \frac{\partial G^{(1)}_j}{\partial x} \left( \sum _k \lambda _{jk} G^{(2)}_k\right) + \sum _{j} \frac{\partial H^{(1)}_j}{\partial x} \left( \sum _k \mu _{jk} H^{(2)}_k\right) ,\\&G^{(1)}_j =0 \text { for } j=1,\ldots ,m_1,\\&H^{(1)}_j =0 \text { for } j=1,\ldots ,n_1. \end{aligned}$$

Defining $\lambda ^{(1)}_j=\sum _k \lambda _{jk} G^{(2)}_k$ and $\mu ^{(1)}_j=\sum _k \lambda _{jk} H^{(2)}_k$, we see that $x^*$ satisfies Lagrange conditions for the optimization problem max *F*(*x*) subject to the generators of $J_1+K_1$. If $G^{(2)}_k(x^*)=0$ for all *k* and/or $H^{(2)}_k(x^*)=0$ for all *k*, then we get other combinations $J_1+K_2$, $J_2+K_1$ or $J_2+K_2$. $\square$

Lemma 1 suggests that if *S* is a singleton in Step 2 of Algorithm 1, then we can replace the ideal $L_S$ of Lagrange conditions for $I_S$ in Step 2 of Algorithm 1 by the ideals of Lagrange conditions for minimal primes of $I_S$. If $S=\{i_1,\ldots ,i_{|S|}\}$, then $I_S=I_{\{i_1\}}+\ldots +I_{\{i_{|S|}\}}$. Hence by Lemmas 1 and 2, we can replace the ideal $L_S$ by the ideals of Lagrange conditions for the sum of minimal primes of $I_{\{i_j\}}$, where $1\le j \le |S|$.

### Remark 3

As discussed in Section 3.2 of the supplementary material to Gross et al. (2016), one can ignore all the components where one of the constraints is $x_k=0$ or the sum of some variables is zero. If one of the variables is zero, then the value of the log-likelihood function is $-\infty$. If the sum of some variables is zero, then all of them have to be zero, because none of them can be negative.

We summarize the results of Lemmas 1, 2 and Remark 3 in Algorithm 2. The output of Algorithm 2 is a list of ideals. For each of the ideals consider the optimization problem where equation constraints are given by the generators of the ideal. The ideals generated by the Lagrange conditions for the optimization problems can be used in Step 2 of Algorithm 1 instead of the ideals $L_S$ for every $S \subseteq [m]$. 
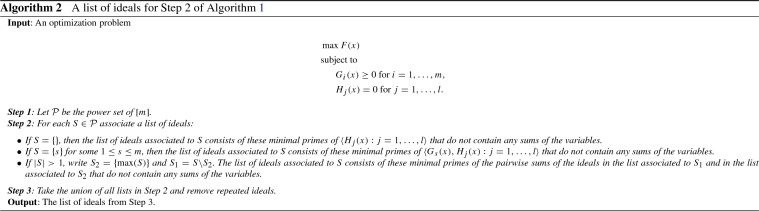


### Remark 4

In practice, it is crucial to know the degrees of the ideals $L_S$ of Lagrange conditions. We recall that these degrees are also known as ML degrees. Although in theory, polynomial homotopy continuation finds all solutions of a system of polynomial equations with probability one, in practice, this can depend on the settings of the program. Without knowing the ML degree, there is no guarantee that any numerical method finds all critical points. For the CFN model on $K_{1,3}$, we experimented with Bertini Bates et al. (2006), NumericalAlgebraicGeometry package in Macaulay2  Leykin (2011) and PHCpack  Verschelde (1999). We ran these three programs with default settings to find the critical points of the log-likelihood function on the Zariski closure of the CFN model on $K_{1,3}$. For our example, only PHCpack found all 92 critical points discussed in Example 4.

### Example 5

We aim to compute the MLE for the CFN model on $K_{1,3}$ and the data vector $\mathbf u =(17, 5, 27, 5, 16, 5, 19, 6)$. This data vector is obtained by generating 100 samples from the distribution inside the CFN model with rate parameters

$$\begin{aligned}&\psi ^{(e_{\text {root}})}=(-0.25,0.25),\psi ^{(e_1)}=(-0.75,0.75),\\&\psi ^{(e_2)}=(-50,50),\psi ^{(e_3)}=(-0.25,0.25). \end{aligned}$$

**Fig. 1:**
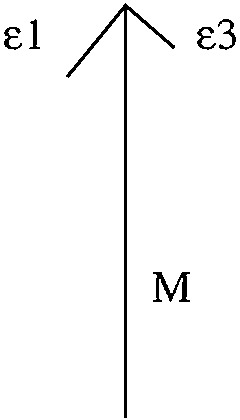
The tree in Example 5 has two edges with short branch lengths $\epsilon _1$ and $\epsilon _3$, one edge with a long branch length *M* and the root distribution is very close to the uniform distribution

The corresponding tree is depicted in Figure 1. It has two short edges, one long edge and the root distribution is very close to the uniform distribution.

To find the MLE, we have to consider three different optimization problems corresponding to the three different cases in Example 2. In each of the cases, we relax the implicit characterization given in Example 2 by replacing strict inequalities with non-strict inequalities. Specifically, in the first case, the polynomials $G_i$ are given by the left-hand sides of the inequalities (9)–(19) and the polynomials $H_j$ are given by the left-hand sides of Eqs. (5)–(8); in the second case, the polynomials $G_i$ are given by the left-hand sides of the inequalities (24)–(34) and the polynomials $H_j$ are given by the left-hand sides of Eqs. (20)–(23); in the third case, the polynomials $G_i$ are given by the left-hand sides of the inequalities (41), (43), (44), (46)–(48) and the polynomials $H_j$ are given by the left-hand sides of Eqs. (35)–(40), (42) and (45). We apply the modified version of Algorithm 1 that uses the output of Algorithm 2 in Step 2. It is enough to run Algorithm 2 and Step 2 of Algorithm 1 for the first optimization problem only as the polynomials $G_i$ and $H_j$ are the same for the first two optimization problems; in the third optimization problem there is one polynomial less and some polynomials $G_i$ are among polynomials $H_j$, but all ideals considered in Algorithm 2 and Step 2 of Algorithm 1 for the third optimization problem are among the ideals for the first optimization problem. In Step 3 we have to check whether elements satisfy $G_i(x) \ge 0$ and $H_j(x)=0$ for any of the three optimization problems. The code for this example can be found at the link:

https://github.com/kaiekubjas/phylogenetics

As a result, we obtain 44 ideals summarized in Table 1. The first row of this table corresponds to the Zariski closure of the CFN model on $K_{1,3}$. It has degree 92 which agrees with the ML degree 92 computed in (Hosten et al. 2005, Example 14). However, to find the MLE one has to consider critical points of the likelihood function in the interior and on all the boundary components, in total 167 of them. We compute all the 167 complex critical points using numerical algebraic geometry software PHCpack. Out of the 167 complex critical points 99 are real and 51 are positive. We list the seven points among them that have the highest value of the log-likelihood function in Table 2.

**Table 1:** Table summarizing different boundary components

Dim *I*	Degree *L*	# of ideals
5	92	1
4	9	4
4	1	8
3	1	24
2	1	6
1	1	1
Total	167	44

**Table 2:** Critical points with the highest values of the log-likelihood function

p	$l_{u}$	MLE
(.183, .051, .256, .055, .147, .053, .204, .052)	$-$ 188.451	No
(.183, .049, .243, .065, .156, .042, .207, .055)	$-$ 188.722	No
(.191, .053, .243, .042, .156, .065, .199, .051)	$-$ 188.803	No
(.165, .05, .23, .055, .165, .05, .23, .055)	$-$ 188.927	No
(.17, .045, .225, .06, .17, .045, .225, .06)	$-$ 189.042	No
(.174, .059, .221, .046, .174, .059, .221, .046)	$-$ 189.303	No
(.22, .05, .22, .05, .175, .055, .175, .055)	$-$ 189.488	Yes

The first six critical points in Table 2 satisfy

$$\begin{aligned} p_{000} - p_{001} + p_{010} - p_{011} + p_{100} - p_{101} + p_{110} - p_{111} > 0 \end{aligned}$$

and

$$\begin{aligned} p_{000} + p_{001} - p_{010} - p_{011} + p_{100} + p_{101} - p_{110} - p_{111} < 0. \end{aligned}$$

Hence these critical points are not in the CFN model on $K_{1,3}$ as in all three cases in Example 2, the two linear inequalities are satisfied with the same sign.

The critical point with the seventh highest log-likelihood value is in the image of the following parameters:

$$\begin{aligned}&\psi ^{(e_{\text {root}})}=(-0.192,0.192),\psi ^{(e_1)}=(-1.071,1.071),\\&\psi ^{(e_2)}=(-\infty ,\infty ),\psi ^{(e_3)}=(-0.080,0.080). \end{aligned}$$

This implies that the MLE for the CFN model on $K_{1,3}$ and the data vector $\mathbf u =(17, 5, 27, 5, 16, 5, 19, 6)$ does not exist—the global maximum of the log-likelihood function is achieved when we allow one of the parameters to go to infinity. Strictly speaking this statement is true for the set of points in the model that satisfy CRCQ. We believe that for random data the global maximum will satisfy CRCQ with probability one. When we run the same optimization problem in Mathematica, then we get a solution with similar value for the log-likelihood function and all parameters besides $\psi ^{(e_2)}$, which is equal to $\psi ^{(e_2)}=(-8.120,8.120)$. Without having the implicit description of the CFN model on $K_{1,3}$ and using numerical algebraic geometry to study the MLE, it would be very difficult to say that the MLE does not exist.

### Remark 5

In Example 5, we chose the rate parameters of the true data generating distribution such that the joint leaf probabilities of this distribution would be close to the boundary of the model. In particular, the Fourier leaf probabilities $q_{010},q_{011},q_{110},q_{111}$ are almost zero. We recall that the semialgebraic description of the CFN model includes strict inequalities $\mathbf q >0$. The global maximum of the likelihood function on the closure of the CFN model on $K_{1,3}$ satisfies $q_{010}=q_{011}=q_{110}=q_{111}=0$. Since this global maximum is not in the model, the MLE does not exist. We expect the similar phenomenon that if our true data generating distribution is close to the boundary, then the MLE does not exist to happen with nonzero probability. In particular, if the normalized data vector lies on the part of the boundary that is not in the model, then we know that the MLE does not exist.

## Proof of Proposition 3

### Proof

The constraints in items (i) and (iv) are obtained, because the corresponding maps are invertible linear transformations. We will prove that the constraints in items (ii) and (iii) are correct.

### Lemma 3

The image of $\{\check{\psi }^{(e)}:\check{\psi }^{(e)}(0)=0, (K^{-1} \check{\psi }^{(e)})(g_1)=(K^{-1} \check{\psi }^{(e)})(g_2) \text {whenever} L(g_1)=L(g_2) \text { and } (K^{-1} \check{\psi }^{(e)})(g) \ge 0 \text {for all nonzero } g \in \mathcal {G}\}$ under the map (2) is described by the equation and inequalities in item (ii).

### Proof

The inequalities $\check{f}^{(e)}(g) > 0$ hold because the image of the exponentiation map is positive. The inequality $a^T x \ge 0$ is equivalent to $e^{a^T x} \ge 1$, and since $e^{a^T x}=(e^x)^{a^T}$, it is also equivalent to $(e^x)^{a^T} \ge 1$. Similarly, the equation $a_1^T x =a_2^T x$ is equivalent to $(e^x)^{a_1^T}=(e^x)^{a_2^T}$. Hence the equations and inequalities for $\{\check{\psi }^{(e)}\}_{e \in E}$ imply $\check{f}^{(e)}(0)=1$, $(\check{f}^{(e)})^{(K^{-1})_{g_1,:}}=(\check{f}^{(e)})^{(K^{-1})_{g_2,:}}$ whenever $L(g_1)=L(g_2)$ and $(\check{f}^{(e)})^{(K^{-1})_{g,:}} \ge 1$ for all nonzero $g \in \mathcal {G}$. $\square$

### Lemma 4

The image of $\{\check{f}^{(e)}:\check{f}^{(e)}(0)=1,(\check{f}^{(e)})^{(K^{-1})_{g_1,:}}=(\check{f}^{(e)})^{(K^{-1})_{g_2,:}} \text {whenever} L(g_1)=L(g_2), (\check{f}^{(e)})^{(K^{-1})_{g,:}} \ge 1 \text { for all nonzero } g \in \mathcal {G} \text { and } \check{f}^{(e)}(g) > 0 \text { for all } g \in \mathcal {G}\}$ under the map (3) is described by the equations and inequalities in item (iii).

Lemma 4 is very similar to (Matsen 2009, Proposition 3.5); however, for the sake of completeness, we will give a proof here. We also include the inequalities $\mathbf{q} > 0$ that do not appear in (Matsen 2009, Proposition 3.5).

### Proof

The inequalities $\mathbf{q} > 0$ are clearly valid inequalities. We will show that we do not have to additionally consider the inequalities $\check{f}^{(e)} > 0$ to construct inequalities for $\mathbf{q}$. Assume there is $\{\check{f}^{(e)}\}_{e \in E}$ with not all entries positive that satisfies all other inequalities in item (ii) and maps to $\mathbf{q} > 0$. We claim that $\{|\check{f}^{(e)}|\}_{e \in E}$ also satisfies the same inequalities in item (ii) and it clearly maps to the same $\mathbf{q}$. Indeed, since the inequalities are of the form $(\check{f}^{(e)})^{2(K^{-1})_{g,:}} \ge 1$, it means that in the product $(\check{f}^{(e)})^{2(K^{-1})_{g,:}}$ minus signs cancel out and hence the absolute values give the same product.

The map (3) is an isomorphism between $\{\check{f}^{(e)}:\check{f}^{(e)}>0\}$ and the positive part of the Zariski closure of the image of $\{\check{f}^{(e)}:\check{f}^{(e)}>0\}$ under the map (3). Indeed, let the composition of the maps in Propositions 1 and 2 with the map (3), map $\mathbf{q}$ to $\{\sqrt{\frac{\mathbf{q}^{a_g}}{\mathbf{q}^{b_g}}}\}_{g \in \mathcal {G}^n}$ for some vectors $\mathbf{a}_g,\mathbf{b}_g \in \mathbb {R}^{\mathcal {G}^n}$. Since $q_g=\sqrt{\frac{\mathbf{q}^{a_g}}{\mathbf{q}^{b_g}}}$, or equivalently $q_g^2 \mathbf{q}^{b_g}=\mathbf{q}^{a_g}$, for all $\mathbf{q}$ in the image, the same equation must be satisfied for all elements in the Zariski closure of the image. Moreover, $\sqrt{\frac{\mathbf{q}^{a_g}}{\mathbf{q}^{b_g}}}$ is well-defined on the positive part of the Zariski closure, hence we have the isomorphism. It follows that on the positive part of the Zariski closure we get the inequalities for $\mathbf{q}$ by substituting expressions for $[\check{f}^{(e)}]^2$ to inequalities $(\check{f}^{(e)})^{2(K^{-1})_{g,:}} \ge 1$. $\square$

This completes the proof that the equations and inequalities in items (i)–(iv) are correct. $\square$
